# Impact of Low Skeletal Muscle Mass on Complications and Survival for Gastric Cancer: A Propensity Score Matching Analysis

**DOI:** 10.3389/fsurg.2022.901142

**Published:** 2022-05-11

**Authors:** Zhen Fang, Liang Shang, Leping Li

**Affiliations:** ^1^ Department of General Surgery, Xuanwu Hospital, Capital Medical University, Beijing, China; ^2^ Department of Gastrointestinal Surgery, Shandong Provincial Hospital Affiliated to Shandong First Medical University, Jinan, Shandong, China

**Keywords:** gastric cancer_1_, psoas muscles index (PMI)_2_, prognosis_3_, complications_4_, propensity score matching analysis_5_

## Abstract

**Background:**

Cancer is a major disease burden to society. Increasing evidence has indicated that low skeletal muscle mass is linked with cancer prognosis. The purpose of the study is to determine the impact of preoperative low skeletal muscle mass (LSMM) on complications and survival of patients who undergo laparoscopic gastrectomy for gastric cancer (GC).

**Methods:**

This study retrospectively collected patients undergoing laparoscopic gastrectomy for GC between January 2017 and December 2018. Tumor staging was performed according to the American Joint Committee on Cancer 8th edition. The third lumbar psoas index (PMI) was assessed by computed tomography (CT) within 15 days before surgery. Postoperative complications were classified according to Clavien-Dindo classification and dichotomized into none vs any (Clavien-Dindo score, ≥1). Using propensity score matching (1:1) to obtain 2 well-balanced cohorts for available variables influencing clinical outcomes, comparing the postoperative complications and 3-year overall survival (OS) between LSMM group and non-LSMM group.

**Results:**

A total of 386 patients, 226 were matched for analyses. The average patient age was 57.31 ± 10.33 years; 75.65% (*n* = 292) were men and 24.35% (*n* = 94) were women. A total of 249 (64.51%) patients were diagnosed with LSMM. Compared with the non-LSMM group, the LSMM group manifested significantly shorter 3-year OS (58.14% vs 71.95%, *p *= 0.034). However, the incidence of postoperative complications was no difference between two groups after matching. After stratification based on the pT stage of the tumor, statistically significant difference in the 3-year OS rates of the advance GC cohort between the two groups were observed.

**Conclusions:**

LSMM predicts a poor prognosis for patients with advance GC and it is not associated with postoperative complications.

## Introduction

Gastric cancer (GC) is one of the most common gastrointestinal malignancies worldwide. According to the latest global cancer statistics, GC is the 5th most common malignant tumor and the 4th leading cause of cancer-related mortality (1–3). With the rapid development of minimally invasive surgery, laparoscopic gastrectomy has become the first choice for the treatment of GC without metastasis. However, it is associated with the occurrence of postoperative complications, tumor recurrence and even death (4, 5).

Skeletal muscle depletion initially defined as the progressive loss of skeletal muscle mass as a result of the aging process (6). Recently, it has garnered much attention because of its association with adverse outcomes in cancer patients, such as postoperative complications, poor prognostic, and death (7–14). However, some studies have also found that skeletal muscle depletion is very common in patients undergoing surgery for GC, but is not associated with worse outcomes (15). Therefore, we conducted this study to clarify the association of skeletal muscle depletion with adverse outcomes.

Propensity score analysis is a statistical technique commonly used to evaluate the efficacy of clinical interventions in observational studies (16, 17). Propensity score matching (PSM) is to match individuals with the same or similar propensity scores in the control group and the treatment group, so as to balance the covariates between the groups and reduce the selection bias of the treatment group. The propensity scoring method can make observational research achieve the effect of randomization without excessive stratification and over-matching. PSM can provide more reliable evidence. So far, there is no research using PSM to analyze the relationship between skeletal muscle mass and the outcome of GC after gastrectomy.

In this study, we focused on skeletal muscle mass by measuring bilateral psoas muscles mass using computed tomography (CT) scan before surgery. The primary aim of the study was to ascertain the effect of preoperative low skeletal muscle mass (LSMM) on postoperative complications and OS rate via using PSM.

## Materials and Methods

### Patients

Patients who underwent laparoscopic gastrectomy for primary GC at our hospital between January 2017 and December 2018 were enrolled in this retrospective study. The inclusion criteria: (1) patients who were pathologically diagnosed as primary GC; (2) patients who were administrated for the primary diagnosis and were therapy naive; (3) patients who were performed laparoscopic gastrectomy. Patients were excluded if (1) they had chemoradiotherapy, targeted therapy before surgery; (2) they had identified metastasis before surgery; (3) CT examination was not performed within 15 days before surgery; (4) their clinical data and follow-up data were incomplete or non-detailed. The study was approved by the Ethics Committee of Shandong Provincial Hospital.

### Data Acquisition

All relevant data were retrospectively collected from the hospital database, including age, sex, body mass index (BMI), nutrition risk screening 2002(NRS2002), tumor grade, TNM stage, albumin, total protein, hemoglobin, lymphocyte percentage (LYM%), platelets, white blood cell (WBC), type of resection, comorbidities and 1-, 3-year follow-up records.

### Skeletal Muscle Mass Analysis

Computed tomography (CT) images of the L3 level on two consecutive transverse sections was used to assess psoas muscle mass. To calculate the PMI, bilateral psoas muscles mass assessed by CT scan is divided by the square of the patient’s height. LSMM was accepted when the PMI was 6.36 cm^2^/m^2^ or less for men and 3.92 cm^2^/m^2^ or less for women (cut-off values determined in Asian populations (18)). All CT images were analyzed by two trained observers. The reader was blinded to each patient’s diagnosis and clinical state. The third lumbar psoas index (PMI) was assessed by computed tomography (CT) within 15 days before surgery.

### Variables and Definitions

According to the international classification of gastric cancer, early gastric cancer is defined as a lesion confined to the mucosa or submucosa and presence or absence of regional lymph node metastasis, while advanced gastric cancer is defined as T2–T4 cancer without distant metastasis. TNM staging was based on the American Joint Committee on Cancer (AJCC) 8th edition. The surgical methods of gastrectomy include total gastrectomy and subtotal gastrectomy. Subtotal gastrectomy includes proximal gastrectomy and distal gastrectomy. In the study, postoperative complications were defined as surgical related adverse events within 30 days after surgery. Postoperative complications were classified according to Clavien-Dindo classification and dichotomized into none vs any (Clavien-Dindo score, ≥1).

### Statistical Analysis

The statistical analyses were performed using IBM SPSS Statistics 26 in this study. Continuous variables were presented as mean ± standard deviation (SD). Continuous variables were compared between groups using the independent *t*-test or Mann-Whitney *U*-test. Categorical variables were analyzed by *χ*^2^ test or Fisher’s exact test. Patients in the cohorts of LSMM and non-LSMM were propensity score-matched at a 1:1 ratio using the Nearest Neighbor Matching approach with a caliper = 0.2. Propensity Score Matching based on age, preoperative BMI, albumin, NRS2002, LYM%, WBC. A univariate and a multivariate sensitivity analysis were also performed. Kaplan–Meier method and Log-rank test were performed to conduct survival analyses and evaluate differences in survival time, respectively. Values of *p* < 0.05 were considered statistically significant. The assessment of propensity score matching is also shown (Supplementary Figure S1).

## Results

### Population

**Figure 1:**
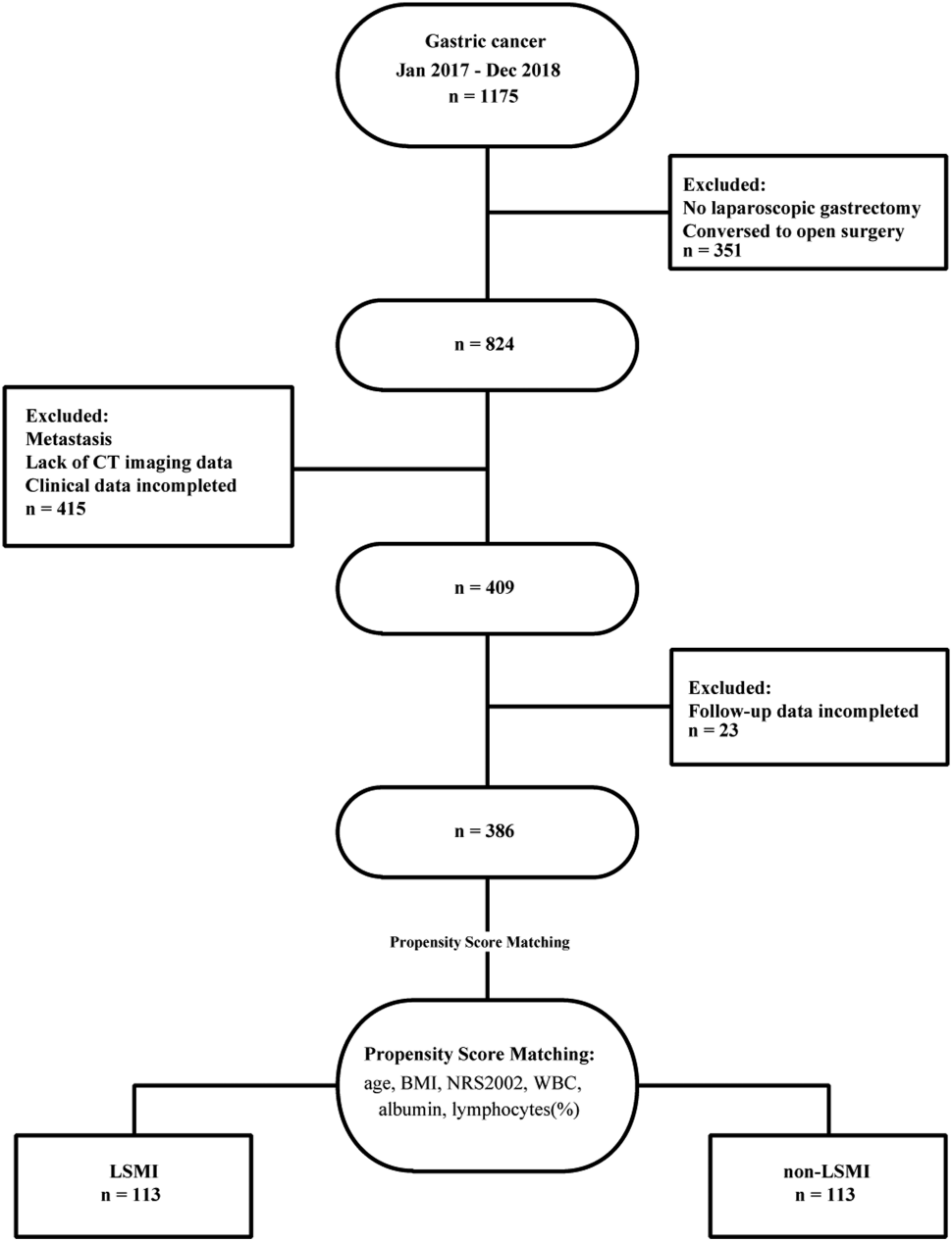
Flow diagram describing the patient matching process.

From January 2017 to December 2018, 386 patients were enrolled and 789 were excluded in the study. Figure 1 is a flow diagram describing the patient selecting and matching process. A total of 351 patients were excluded because they did not undergo laparoscopic gastrectomy. 415 patients were excluded due to incomplete clinical data, no preoperative abdominal CT or distant metastasis. Follow-up data were incomplete for 23 of the remaining 409 patients. The average patient age was 57.31 ± 10.33 years; 75.65% (*n* = 292) were men and 24.35% (*n* = 94) were women. A total of 249 (64.51%) patients were diagnosed with LSMM (Table 1).

**Table 1:** Characteristics of the study patients.

Characteristic	Value
Age (yr)	57.31 ± 10.33
Sex	
Male	292
Female	94
BMI (kg/m^2^)	24.01 ± 2.81
LSMM	
Yes	249
No	137
Smoker	174
Drinker	176
NRS 2002 score	
<3	209
≥3	177
Comorbidities	
Hypertension	90
Diabetes	49
Chronic kidney disease	21
Chronic lung disease	29
Tumor grade	
I–II	126
III	260
TNM Stage	
Stage I–II	178
Stage III	208
T Stage	
Early	95
Advance	291
Type of resection	
Total gastrectomy	62
Subtotal gastrectomy	324
Laboratory indicators	
Albumin (g/L)	41.02 ± 4.31
Total protein (mg/L)	67.12 ± 5.74
Hemoglobin (g/L)	132.03 ± 21.81
Lymphocytes (×10^9^/L)	1.65 ± 0.52
Lymphocytes (%)	29.69 ± 8.63
Platelets (×10^9^/L)	248.36 ± 70.93
WBC (×10^9^/L)	5.73 ± 1.58

*NRS 2002, nutrition risk screening 2002; BMI, body mass index; LSMM, low skeletal muscle mass.*

### Comparison of the Clinical and Pathological Data Between the LSMM and non-LSMM Groups Before and After Propensity Score Matching

Patients’ demographic characteristics and clinical features are listed in Table 1. Before propensity-matching, there were statistically significant differences in the age (*p *< 0.0001), BMI (*p *< 0.0001), NRS2002 (*p *< 0.0001), albumin (*p *< 0.0001), LYM% (*p *= 0.01) and WBC (*p *= 0.006) between the two groups, but no significant differences were observed in the sex, tumor histological grade, pT Stage, pTNM Stage, comorbidities or type of resection (all *p *> 0.05) (Table 2). After the propensity score matching ratio was set to 1:1, the clinical and pathological data of 113 patients in the LSMM group were found comparable with 113 patients in the non-LSMM group (*p *> 0.05 between all variables) (Table 2).

**Table 2:** Patient demographic characteristics and clinical features before and after propensity score matching.

Parameter	Before Propensity-matching		*p* value	After Propensity-matching		*p* value
	LSMM (*n* = 249)	non-LSMM (*n* = 137)		LSMM (*n* = 113)	non-LSMM (*n* = 113)	
Age (yr)	59.47 ± 9.53	53.39 ± 10.65	**<0.0001**	55.42 ± 9.67	54.55 ± 10.07	0.505
Sex			0.736			0.656
Male	187(75.1%)	105(76.64%)		80(70.8%)	83(73.45%)	
Female	62(24.9%)	32(23.36%)		33(29.2%)	30(26.55%)	
BMI (kg/m^2^)	23.37 ± 2.55	25.25 ± 2.88	**<0.0001**	24.46 ± 2.35	24.61 ± 2.67	0.656
Smoker	117(46.99%)	57(41.61%)	0.309	52(46.02%)	46(40.71%)	0.421
Drinker	108(43.37%)	68(49.64%)	0.237	51(45.13%)	52(46.02%)	0.894
NRS 2002 score			**<0.0001**			0.89
<3	117(46.99%)	92(67.15%)		72(63.72%)	73(64.6%)	
≥3	132(53.01%)	45(32.85%)		41(36.28%)	40(35.4%)	
Comorbidities						
Hypertension	52(20.88%)	38(27.74%)	0.128	21(18.58%)	30(26.55%)	0.152
Diabetes	30(12.05%)	19(13.87%)	0.607	12(10.62%)	15(13.27%)	0.538
Chronic kidney disease	15(6.02%)	6(4.38%)	0.495	8(7.08%)	7(6.2%)	0.789
Chronic lung disease	22(8.84%)	7(5.11%)	0.184	4(3.54%)	6(5.31%)	0.748
Tumor grade			0.772			
I–II	80(32.13%)	46(33.58%)		39(34.51%)	36(31.86%)	
III	169(67.87%)	91(66.42%)		74(65.49%)	77(68.14%)	
TNM Stage			0.214			0.69
Stage I–II	109(43.78%)	69(50.37%)		54(47.79%)	57(50.44%)	
Stage III	140(56.23%)	68(49.64%)		59(52.21%)	56(49.56%)	
Type of resection			0.384			0.597
Total gastrectomy	43(17.27%)	19(13.87%)		21(18.58%)	18(15.93%)	
Subtotal gastrectomy	206(82.73%)	118(86.13%)		92(81.42%)	95(84.07%)	
Laboratory indicators						
Albumin (g/L)	40.40 ± 4.18	42.15 ± 4.34	**<0.0001**	41.39 ± 4.16	41.75 ± 4.12	0.529
Total protein (mg/L)	66.75 ± 5.24	67.78 ± 6.54	0.114	67.69 ± 5.30	67.38 ± 6.71	0.704
Hemoglobin (g/L)	131.29 ± 21.48	133.36 ± 22.48	0.375	133 ± 22.12	132.83 ± 22.79	0.955
Lymphocytes (×10^9^/L)	1.64 ± 0.55	1.66 ± 0.46	0.685	1.63 ± 0.55	1.67 ± 0.47	0.532
Lymphocytes (%)	28.91 ± 9.20	31.13 ± 7.33	**0.01**	30.48 ± 9.23	30.52 ± 7.11	0.964
Platelets (×10^9^/L)	249.48 ± 75.61	246.33 ± 62.04	0.677	245.12 ± 70.10	245.33 ± 61.96	0.982
WBC (×10^9^/L)	5.88 ± 1.71	5.45 ± 1.29	**0.006**	5.48 ± 1.50	5.56 ± 1.32	0.695

*NRS 2002, nutrition risk screening 2002; BMI, body mass index; LSMM, low skeletal muscle mass.*

*The bold values in P value: P < 0.05.*

### Univariate and Multivariate Analysis of Risk Factors for Postoperative Complications

The distribution of postoperative complications is listed in Table 3. Univariate and multivariate analysis identified the following as prognostic factors for postoperative complications: albumin (*p *= 0.036), hemoglobin (*p *= 0.03), smoker (*p *= 0.014). There were no statistically significant differences in the complications between the LSMM group and non-LSMM group after propensity score matching (*p *= 0.654) (Table 4).

**Table 3:** Postoperative complications between patients with LSMM or non-LSMM.

Parameter	Before Propensity-matching		After Propensity-matching	
	LSMM (*n* = 249)	non-LSMM (*n* = 137)	LSMM (*n* = 113)	non-LSMM (*n* = 113)
All complications				
Infectious complications				
Incision infection	4	3	2	3
Intra-abdominal abscess	6	0	2	0
Pneumonia	17	5	7	5
Noninfectious complications				
Anastomotic leakage	7	1	0	1
Duodenal stump leakage	2	0	0	0
Chylous ascites	2	0	0	0
Gastrointestinal obstruction	6	1	1	1
Bleeding	2	0	0	0

**Table 4:** Univariate and multivariate analysis of risk factors for postoperative complications.

Parameter	Categories	Complications		Univariate analysis			Multivariate analysis		
		Yes (*n* = 22)	No (*n* = 204)	OR	95% CI	*p*-value	OR	95% CI	*p*-value
Sex	Male	18	145	1.832	0.595–5.650	0.286			
	Female	4	59	Reference					
Age(yr)		53.55 ± 8.25	55.14 ± 10.02			0.472			
LSMM	Yes	12	101	1.224	0.506–2.959	0.654	0.922	0.291–2.923	0.89
	No	10	103	Reference			Reference		
BMI (kg/m^2^)		25.29 ± 2.41	24.45 ± 2.51			0.138			0.456
NRS 2002	≥3	22	59			<0.0001			0.995
	<3	0	145						
Drinker	Yes	10	93	0.995	0.411–2.406	0.99			
	No	12	111	Reference					
Smoker	Yes	15	83	3.124	1.221–7.994	0.013	4.111	1.328–12.720	**0.014**
	No	7	121	Reference			Reference		
Diabetes	Yes	2	25	0.716	0.158–3.250	0.653			
	No	20	179	Reference					
Tumor grade	I–II	16	135	1.363	0.510–3.639	0.535			
	III	6	69	Reference					
T Stage	Early	4	54	0.617	0.200–1.905	0.398			
	Advance	18	150	Reference					
TNM Stage	I–II	6	105	0.354	0.133–0.939	0.031	1.675	0.496–5.660	0.406
	III	16	99	Reference			Reference		
Laboratory									
Albumin (g/L)		39.73 ± 4.15	41.77 ± 4.26			0.038			**0.036**
Total protein (mg/L)		65.57 ± 5.21	67.75 ± 6.09			0.108			** **
Hemoglobin (g/L)		132.95 ± 24.45	132.91 ± 22.24			0.993			**0.03**
Platelets (×10^9^/L)		235.14 ± 51.25	246.31 ± 67.42			0.452			
Lymphocytes (×10^9^/L)		1.63 ± 0.60	1.65 ± 0.51			0.856			
Lymphocytes (%)		27.74 ± 6.59	30.80 ± 8.33			0.097			
WBC (×10^9^/L)		5.62 ± 1.43	5.51 ± 1.41			0.718			

*NRS 2002, nutrition risk screening 2002; BMI, body mass index; LSMM, low skeletal muscle mass.*

*The bold values in P value: P < 0.05.*

### Impact of LSMM on 1-, 3-Year OS

**Figure 2:**
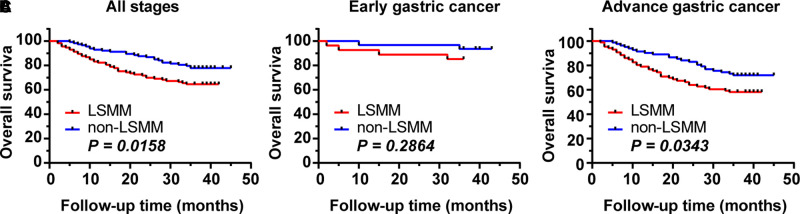
Impact of LSMM on OS. (**A**) in all patients of GC. The 3-year OS rate was 71.24% for all patients, 64.6% and 77.9% for LSMM and non-LSMM groups respectively (*p *= 0.0158). (**B**) in the early GC. The 3-year OS rate were 85.16% and 93.55% for the LSMM and non-LSMM groups respectively (*p *= 0.2864). (**C**) in the advance GC. The 3-year OS rate were 58.14% and 71.95% for the LSMM and non-LSMM groups respectively (*p *= 0.0343).

The median follow-up time of the entire matched cohort was 36 months (range 0–45 months). There are 65 deaths. The 3-year OS rates was 71.24% for all patients. The 1-, 3-year OS rates were 83.2%, 64.6% respectively, for patients with LSMM and 92.9%, 77.9%, respectively, for those non-LSMM. The survival curves for patients of GC with and without LSMM are shown in Figure 2. Patients with LSMM showed a significantly poorer OS than the non-LSMM group (*p* = 0.016, Figure 2A).

After stratification based on the pT stage of the tumor, the 3-year OS rate of the early GC cohort was 89.66%, and no statistically significant difference in the 3-year OS rates between the LSMM (3-year OS, 85.16%) and non-LSMM (3-year OS, 93.55%) were found (*p *= 0.286, Figure 2B). However, the 3-year OS rate of the advance GC cohort was 64.89%, and statistically significant difference in the 3-year OS rates between the two groups were observed (58.14% vs 71.95%, *p *= 0.034, Figure 2C).

## Discussion

The study demonstrated that the 3-year OS rate of patients with LSMM was 64.6%, which was significantly poorer than those with non-LSMM (77.9%) (*p *= 0.016) and that LSMM was an independent risk factor for overall survival in patients with GC. Furthermore, preoperative LSMM may be particularly useful in advance GC. However, the results in our study showed that LSMM is not associated with the incidence of postoperative complications.

Several clinical studies have reported that LSMM might be an unfavorable factor for the short-time outcomes and prognosis (19–22), but some existing have shown contrasting results (23–26). Katsunobu et al. (23) have shown that there is no relationship between preoperative LSMM and postoperative complications. Kuroki, L. M. et al. (25) also indicate that LSMM has no negative impact on postoperative complications or overall survival among endometrial cancer patients. The different conclusions may be due to: 1. Different studies have different diagnostic criteria for low skeletal muscle mass; 2. Different tumor types may lead to different conclusions; 3. Different treatment methods; 4. Different races. In this study, we only included patients who underwent laparoscopic gastrectomy. The diagnostic criteria for LSMM came from a research based on Asian populations, which is suitable for the included population. In addition, the application of PSM analysis makes the research conclusions more reliable. Skeletal muscle mass is an excellent indicator for assessing the physical condition, and it is more objective to reflect the nutritional status of the body than Body Mass Index (BMI).

The reason why people pay attention to skeletal muscle mass is that the change of skeletal muscle mass is closely related to the postoperative outcome. Studies have found that skeletal muscle can truly reflect the nutritional status of the body. Decreased skeletal muscle index may indicate nutritional deficiencies or overconsumption. As a result, patients with reduced skeletal muscle index are less able to withstand greater stress or trauma, such as laparoscopic gastrectomy. For those patients with severe skeletal muscle depletion before surgery, clinicians should comprehensively evaluate and formulate more scientific treatment strategies, such as nutritional support treatment (27, 28). Studies have shown that nutritional support program and exercise are effective way to improve postoperative outcomes in patients with GC (29).

In this study, we divided patients into early stage and advanced stage GC for analysis. The results showed that there was significant difference in 3-year OS between the LSMM group and the non-LSMM group in advanced GC (*p *= 0.0343), but no difference was found in early GC (3-year OS, 85.16% vs 93.55%, *p = *0.2864). The initial hypothesis of the study was that the two groups can show differences in early and advanced GC. This is an interesting discovery, and no relevant research has been reported so far. We will continue to follow up these patients to further observe the impact of LSMM on the prognosis of patients with early GC.

CT is a routine examination for patients with GC before surgery, so it is feasible to use CT to assess skeletal muscle mass. It will not bring additional economic burden to patients. This study is a single-center retrospective study. The main purpose is to clarify the relationship between preoperative skeletal muscle mass and prognosis in patients with GC. CT was used to assess the preoperative skeletal muscle mass and found high-risk patients. This is of great significance to the scientific diagnosis and treatment of GC patients.

There are some potential limitations in this study. This study is a retrospective single-center study, the data integrity may be insufficient. We plan to conduct a prospective study to further explore the adverse effects of LSMM.

## Conclusions

By using PSM analysis to balance the differences between cofounding variables, the study has proved that LSMM is an unfavorable factor for OS rates, especially in patients with advance GC. However, LSMM is not associated with postoperative complications.

## Data Availability

The original contributions presented in the study are included in the article/Supplementary Material, further inquiries can be directed to the corresponding author/s.
