# Supplementary figures and images for: Impact of Low Skeletal Muscle Mass on Complications and Survival for Gastric Cancer: A Propensity Score Matching Analysis
Source: Front Surg. 2022 May 11;9:901142. doi: 10.3389/fsurg.2022.901142 (PMC9130631; doi:10.3389/fsurg.2022.901142)

# Supplemental Figure1

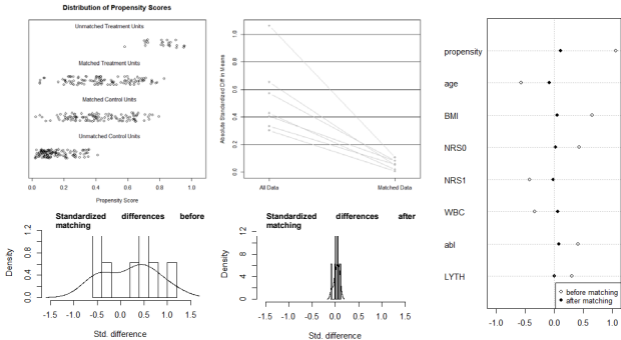

Supplement: Supplementary file 1 [file Data_Sheet_1.pdf]
